# Overinvestment in selected Central and Eastern European countries: Production and economic effects

**DOI:** 10.1371/journal.pone.0251394

**Published:** 2021-05-07

**Authors:** Krzysztof Piotr Pawłowski, Wawrzyniec Czubak, Jagoda Zmyślona, Arkadiusz Sadowski

**Affiliations:** Department of Economics and Economic Policy in Agribusiness, Faculty of Economics, Poznan University of Life Sciences, Poznan, Poland; Szechenyi Istvan University, HUNGARY

## Abstract

Farms need to invest in order to earn incomes and maintain their competitive edge. However, the scale and extent of investments must be aligned with resources of other productive inputs, primarily including land, because otherwise there is risk of overinvestment. Since 2004, Central and Eastern European countries have been provided with access to investment support programs; these are non-repayable aid funds which can potentially lead to overinvestment issues. Therefore, this paper attempts to answer the question on the scale of overinvestment in the countries covered. This is all the more important since that problem has rarely been addressed in economic and agricultural research. The study presented in this paper is unique in that the research tasks are based on unpublished Farm Accountancy Data Network (FADN) microdata for 5839 selected Central and Eastern European farms provided by the European Commission’s Directorate-General for Agriculture and Rural Development (DG AGRI). Based on variables relating to the amount of productive inputs and production volumes, the authors developed their own typology of farms which includes the following categories: optimum investment levels (the growth rate of labor productivity is greater than growth in the assets-to-land ratio); relative overinvestment (while labor productivity grows, it does so at a slower rate than the assets-to-land ratio); absolute overinvestment (labor productivity declines while the assets-to-land ratio grows); underinvestment (decline in both labor productivity and the assets-to-land ratio). The authors demonstrated that members of the ‘absolute overinvestment’ group made flagrant mistakes in investment planning and implementation, whereas members of the ‘relative overinvestment’ group did record an improvement in labor productivity which ultimately can be considered a positive outcome. Underinvested farms were a very small minority in each country. In addition to filling a gap in the methodology for determining agricultural overinvestment, this paper also indicates the scale of that issue in Central and Eastern European countries. This study may be of importance both to farms (as guidelines for investment planning) and to agricultural policymakers who develop investment support programs.

## Introduction

Due to its specific production characteristics related to macro- and microeconomic conditions [1], agriculture is believed to be the main determinant of self-sufficiency in food, protection of natural resources (with main focus on land), rural development and socio-cultural benefits [2]. It also is a part of landscape; for instance, in the European Union (EU), agriculture is the main user of land which accounts for over 47% of total territory [3]. In 2018, the world had a population of 7.6 billion. That number is forecasted to grow, with the greatest increase expected to be recorded in Asian regions such as China, India and Pakistan [4]. Although the Malthusian theory is not supported by how the socioeconomic system evolves, it is worth being referenced to because of its importance in the development of economic theory. It is based on the thesis that population grows exponentially while food production grows arithmetically [5, 6]. Research that identifies links between food, land and population continues to have an effect on how agricultural policy is formulated by international development decision-makers [7, 8]. Also, the considerable increase in the future demand for food will require agricultural production to be intensified in a sustainable way, so that environment may be protected and climatic impacts may be minimized when fulfilling economic functions [9, 10]. Unfortunately, climate change often has a knock-on effect and translates into a number of environmental, agricultural and social consequences while making these matters interdependent [11–15]. Hence, there is need for sustainable development of the agricultural sector in a way to ensure environmental security. Nevertheless, as regards agriculture, human progress means a gradual substitution of labor by capital [16], ultimately leading to growth in farm size, reduction in farm numbers, and less people being employed in agricultural production. These phenomena take place provided that capital is supplied both at sector and farm level as part of investment processes. Investments are of vital importance not only to the continued existence of single operators, for instance due to the effect they have on incomes [17], but also to food security which, in today’s world, may be only be ensured through the use of state-of-the-art technological and organizational solutions. Viewed in the long run, food security requires that environmental sustainability and production growth be ensured at the same time [18]. Hence, it is essential to modernize agriculture which generally means attaining progress through improvements in productive, organizational, technical and technological inputs [19, 20]. Furthermore, agricultural progress has an effect on the economy [21].

Numerous studies point to need for agricultural investments because an improvement in agricultural performance is, for obvious reasons, the basis for an increase in food production volumes [22]. The extent to which humans intervene in the food production process to ensure food security (which includes enough food for everyone, access to nutritious food and providing a food guarantee) [23, 24] is usually measured with the amount of investment expenditure [25, 26]. However, no country managed to alleviate poverty and contribute to improvements in food security based on agriculture alone. Nevertheless, investments in agricultural assets provide a framework for reducing poverty in the long run, increase land productivity and reinforce environmental protection measures [27, 28]. Another essential aspect is institutional and industrial development [22] which ultimately also has an impact on the increase in and rationality of agricultural investments.

Nevertheless, one should be aware of the threats involved in the investment process which takes on a particular dimension in the agriculture. A frequent problem is that the importance of investments is underestimated. This is due to the particularities of agriculture which requires an intense, though relatively short, use of fixed assets because of production seasonality. Investments can be economically unviable which means the economic operator suffers a loss in the long term. When attempting to estimate investment viability, it can be noted that excessive investments (which primarily mean a mismatch between investment level and production scale) ultimately result in operational dysfunctions, including overinvestment. That process is manifested by a “higher capital-to-land ratio” [29], eventually causing a reduction in production efficiency. When considering that problem on an economy-wide or a per-sector basis, it can be concluded that overinvestment results in excess production [30].

In order to identify the extent of agricultural overinvestment, focus must be placed on inefficiencies which occur when the assets-to-labor ratio grows while labor productivity declines [31]. Therefore, this paper addressed the problem of agricultural overinvestment defined as a condition where long-term investments are excessively high compared to the production potential (mainly including land resources) and ultimately become economically unviable [32]. Another method of identifying overinvestment in a publicly subsidized agriculture regime is by calculating the rate of return on public investments in agriculture [33]. The opposite situation (underinvestment) may also occur; this was the case in China when the rates of return on capital investments in agricultural production were much higher than in urban sectors which could be indicative of agricultural underinvestment [34]. As this paper focuses on a microeconomic approach to overinvestment, it examined Central and Eastern European farms in terms of their production and economic performance. The pertinence of addressing this topic is justified because overinvestment in the agricultural sector was observed to affect the use efficiency of resources and to result in a high risk of environmental pollution [35]. Furthermore, no such research has yet been carried out. Although a similar study was undertaken in China based on technical efficiency calculations, it was oriented on production only, and did not take account of the degree of labor productivity [34]. This paper focuses on results underpinned by microdata for selected Central and Eastern European (CEE) countries.

Central and Eastern European countries share a common economic history spanning over the last several decades. From the end of World War 2 until late 1980s, they lived under real socialism, a system which can be briefly characterized as the nationalization of a large part of productive inputs and a centrally planned economy. The fall of that system was accompanied by a crisis which ultimately confirmed its economic inefficiency. However, the economic history of agriculture slightly differs between Central and Eastern European countries [36]. In Poland and Slovenia (until early 1990s, it was part of former Yugoslavia, and was therefore covered by all systemic solutions of that country), private ownership of (family-run) farms was essentially maintained; other countries were dominated by large, nationalized (state-owned or cooperative) holdings. Today, these historical events are reflected in the agrarian structure which differs across countries. For instance, large agricultural holdings play a major role in Czech Republic and Slovakia, whereas other countries are dominated by small family farms while recording a quite considerable share of large enterprises which usually are the former national holdings that underwent a transformation process. Such large operators play the smallest role in Poland and Slovenia [37]. Irrespective of differences referred to above, the crisis of the socialist system resulted in agricultural underinvestment in each of these countries. The farms themselves lacked funds to finance modernization processes and could not rely on state aid. After the fall of real socialism, there were little opportunities to change this situation, including because of the financial deficiency of the restructuring economies. Nevertheless, a number of measures were taken according to the capabilities of each country. In mid-1990s, Poland established a soft loan system primarily intended to finance farm modernization [20, 38]. Central and Eastern European farms saw a great improvement in their upgrading and catching-up opportunities when the respective countries joined the European Union in 2004 and 2007. This is when they became eligible for direct payment schemes and dedicated rural development programs. The latter are of particular importance because they are intentionally largely focused on supporting farm investments. The partially non-repayable form of aid contributed to a considerable improvement in the situation of many beneficiaries [39], especially as they often had a lot of catching up to do. Nevertheless, the capacity to implement essential projects at a relatively low cost encouraged the farmers to invest excessive amounts of funds in relation to other productive resources owned, especially land. This was the case despite the existence of formal limits for access to investment support programs (usually referred to as the minimum physical or economic size) and inspection procedures being implemented by competent paying agencies. Obviously, public support is not a necessary condition for overinvestment. However, the ability to access additional funds increases that risk, especially because it alleviates some economic restrictions that should have been taken into account by both the investor and the paying agency. Overinvestment can coexist with underinvestment, a phenomenon which has less to do with the use of funds under the Common Agricultural Policy. It is manifested in a situation where essential projects are either not implemented at all or are implemented to such a small extent that they do not prevent the value of assets from decreasing. This may have diverse reasons, including: the farmer not having a successor; the competitive potential being viewed as insufficient by the manager; or the deficiencies in strategic management. In the context of CAP instruments, this may also include the thresholds of access to aid.

The purpose of the work is to determine the scale of the overinvestment in agriculture in the countries of Central and Eastern Europe, which since 2004 have been competing on the single European market and may benefit from pro-investment programs supporting the European Union. Based on the assumption of the subordinate role of investment activity in relation to the operational one, it attempted to answer the questions about how the size of the investment affects the relations between production factors and economic results of farms.

The paper is organized as follows: Section 2 presents pertinence of the topic. Section 3 describes the unique FADN source data and the research methods employed. Section 4 presents and discusses the key findings. Finally, Section 5 presents the summary, conclusions, relevant political implications and guidelines for further research.

## Materials and methods

Unpublished FADN (Farm Accountancy Data Network) microdata provided by the European Commission’s DG AGRI was used as the source material. The European Farm Accountancy Data Network was established in European Economic Community countries upon initiating the implementation of CAP mechanisms. Note that only commercial farms are monitored by the system. The study presented in this paper is unique in that the research tasks are based on unpublished microdata of selected Central and Eastern European farms. The microeconomic nature of this data makes it also possible to carry out dynamic analyses [40]. Formal guidelines for working with extremely sensitive data are highly restrictive, and therefore this paper only presents aggregated results for no less than 15 farms. The research covered selected Central and Eastern European countries: Bulgaria, Estonia, Hungary, Latvia, Poland and Slovenia. The study period was 2004–2015. The initial year marks the first enlargement of the EU with CEE countries while the last year corresponds to the most recent data from FADN resources. Of all the farms, this study selected only those or which continuous FADN records were available throughout the analysis period (2004–2015 and 2007–2015 for Bulgaria). The number of farms selected in each country is shown in Table 1.

**Table 1:** Number of farms covered by this study (by country).

Bulgaria	Estonia	Hungary	Latvia	Poland	Slovenia
424	241	855	264	3964	82

Source: own calculations based on unpublished EU-FADN–DG AGRI data.

This paper relies on the authors’ own classification method which assumes that increasing the value of farm assets through investments is a reasonable thing to do if it results in a proportional growth in labor productivity. Therefore, overinvestment is defined as a situation where:

- The increase in the value of assets results in a decline in labor productivity, which may be due to high maintenance costs of particular assets (e.g. depreciation). The above is defined as absolute overinvestment.
- Labor productivity grows at a lower rate than the value of assets. This is referred to as relative overinvestment.

Changes in labor productivity were calculated for each farm. Labor productivity was defined as net value added (gross value added less depreciation) less operating and investment subsidies per FTE. Net value added was used (rather than family farm income) because of the need to eliminate the costs of external inputs (paid labor, rents, interest charged on credits) from the calculation in order to unify the economic performance figures of farms which rely on both their own and external productive inputs. The subsidies were removed from the calculation because public aid cannot be regarded as a metric of labor productivity in the economic sense. This can be assumed even if access to certain subsidies involves (at least formally) the need to perform specific actions, such as meeting the cross compliance or greening requirements in the case of payments. However, these actions refer to the production of public goods, and therefore do not have a direct impact on economic performance recorded in the market. Average values were calculated for the first and last three years of the study period in order to determine the changes. This was used as a basis for calculating the index for changes as per the formula below:

$$LP_{t}=\frac{\sum_{t}^{t+2}\left(\frac{SE410‐SE360‐SE406‐SE605}{SE010}\right)}{3}$$ (1)

$$\mathrm{\Delta LP}=(\frac{{\mathrm{LP}}_{2013‐2015}{‐\mathrm{LP}}_{2004‐2006}}{{\mathrm{LP}}_{2004‐2006}})*100\%$$ (2)

where:

*LP*: labor productivity

*SE410*: gross farm income

*SE360*: depreciation

*SE406*: subsidies on investments

*SE605*: total subsidies (excluding on investments)

*SE010*: total labor input (AWU)

Changes in the assets-to-labor ratio were calculated as the next step. The value of fixed assets less the value of land per FTE was used as a metric of the increase in the assets-to-labor ratio. The value of land includes agricultural land, land improvement machinery, permanent crops, quotas and other rights attached (including purchasing costs) and forest land. Production quotas (and other rights attached) received free of charge are not appraised in the balance sheet (only the sales thereof is recorded). The rationale behind the above approach is that overinvestment is a problem which ultimately leads to a mismatch between the farm area and the extent of investments in machinery and buildings. Average values were calculated for the first and last three years of the study period, just like in the case of labor productivity. The index for changes was determined next:

$$ALR_{t}=\frac{\sum_{t}^{t+2}\left(\frac{SE441-SE446}{SE010}\right)}{3}$$ (3)

$$\mathrm{\Delta ALR}=(\frac{ALR_{2013‐2015}{‐\mathrm{ALR}}_{2004‐2006}}{ALR_{2004‐2006}})*100\%$$ (4)

where:

*ALR*: assets-to-labor ratio

*SE441*: total fixed assets

*SE446*: land, permanent crops and quotas

*SE010*: total labor input

As a next step, each farm was attributed to a group based on the overinvestment criterion:

- Absolute overinvestment; this is the case for farms where labor productivity drops while the assets-to-labor ratio grows:
  ΔLP<0∧ΔALR>0 (5)
- Relative overinvestment; this is the case for farms where both labor productivity and the assets-to-labor ratio are on an increase but the increase in the assets-to-labor ratio is smaller than the increase in labor productivity:
  ΔLP>0∧ΔALR>0∧ΔLP<ΔALR (6)
- Underinvestment; this is the case for farms where both labor productivity and the assets-to-labor ratio are on a decline.
  ΔLP<0∧ΔALR<0 (7)
- Optimum investments; this is the case for farms where both labor productivity and the assets-to-labor ratio are on an increase, and labor productivity grows faster than the assets-to-labor ratio:
  ΔLP>0∧ΔALR>0∧ΔLP>ΔALR (8)

Eight indicators were calculated for each group and for each country separately to identify the reasons for overinvestment. Just like in the case of classification ratios, average values were calculated for the first and last three years of the study period for each indicator.

1. Changes in land resources (ha per farm)
  $$I_{1}=\frac{\sum_{t}^{t+2}\left(\frac{\sum_{i=1}^{n}SE025}{n}\right)}{3}$$ (9)
  where:
  *SE025*: total utilized agricultural area
  *n*: number of farms covered by this study (by country)
2. Changes in labor resources (AWU per farm)
  $$I_{2}=\frac{\sum_{t}^{t+2}\left(\frac{\sum_{i=1}^{n}SE010}{n}\right)}{3}$$ (10)
  where:
  *SE010*: total labor input
  *n*: number of farms covered by this study (by country)
3. Changes in capital resources (EUR per farm)
  $$I_{3}=\frac{\sum_{t}^{t+2}\left(\frac{\sum_{i=1}^{n}\left(SE441-SE446\right)}{n}\right)}{3}$$ (11)
  where:
  *SE441*: total fixed assets
  *SE446*: land, permanent crops and quotas
  *n*: number of farms covered by this study (by country)
4. Changes in fixed asset resources in relation to land resources (EUR/ha)
  $$I_{4}=\frac{\sum_{t}^{t+2}\left(\frac{\sum_{i=1}^{n}\left(SE441-SE446\right)}{\sum_{i=1}^{n}SE025}\right)}{3}$$ (12)
  where:
  *SE441*: total fixed assets
  *SE446*: land, permanent crops and quotas
  *SE025*: total utilized agricultural area
  *n*: number of farms covered by this study (by country)
5. Changes in fixed asset resources in relation to labor resources (EUR/AWU)
  $$I_{5}=\frac{\sum_{t}^{t+2}\left(\frac{\sum_{i=1}^{n}\left(SE441-SE446\right)}{\sum_{i=1}^{n}SE010}\right)}{3}$$ (13)
  where:
  *SE441*: total fixed assets
  *SE446*: land, permanent crops and quotas
  *SE010*: total labor input
  *n*: number of farms covered by this study (by country)
6. Changes in farm productivity (EUR per farm)
  $$I_{6}=\frac{\sum_{t}^{t+2}\left(\frac{\sum_{i=1}^{n}SE131}{n}\right)}{3}$$ (14)
  where:
  *SE131*: total output
  *n*: number of farms covered by this study (by country)
7. Changes in cost intensity at farm level (EUR per farm)
  $$I_{7}=\frac{\sum_{t}^{t+2}\left(\frac{\sum_{i=1}^{n}SE270}{n}\right)}{3}$$ (15)
  where:
  *SE270*: total inputs
  *n*: number of farms covered by this study (by country)
8. Changes in Net Value Added (without taking account of operating and investment subsidies (EUR per farm)
  $$I_{8}=\frac{\sum_{t}^{t+2}\left(\frac{\sum_{i=1}^{n}\left(SE410-SE360-SE406-SE605\right)}{n}\right)}{3}$$ (16)
  where:
  *SE410*: gross farm income
  *SE360*: depreciation
  *SE406*: subsidies on investments
  *SE605*: total subsidies (excluding on investments)
  *n*: number of farms covered by this study (by country)
  The index for changes was defined as follows for each of the indicators listed above:
  $$\Delta I=I_{2013-2015}-I_{2004-2006}$$ (17)

## Results

**Fig 1:**
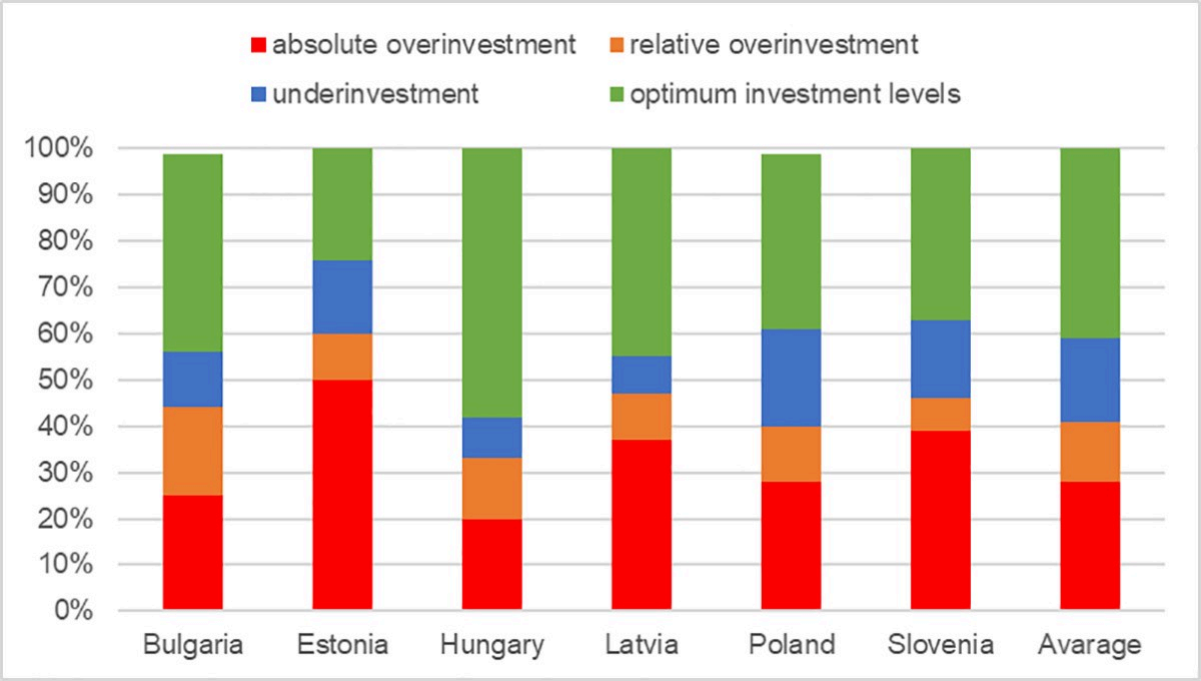
Structure of farms grouped by scale of investment (5). Source: own calculations based on unpublished EU-FADN–DG AGRI data.

The extent of overinvestment and underinvestment differs across Central and Eastern European countries (Fig 1). The relatively greatest number of farms at optimum investment levels can be found in Latvia and the smallest in Estonia (which also is home to the greatest number of farms affected by absolute overinvestment). Relative overinvestment and underinvestment represent a small proportion of cases in each of the countries covered by this study. The main reason for the latter is that after joining the EU, Central and Eastern European countries launched their support programs for farm modernization which essentially consisted in co-financing the investments on a non-repayable basis. Usually, these forms of support were available to a wide group of beneficiaries (the minimum economic size thresholds were set at a relatively low level) which made overinvestment more likely than underinvestment. Moreover, in accordance with the general requirements for co-financing with Union funds, support programs offered only a partial refinancing of investment expenditure. Hence, it was up to farm managers to assess their competitive position and the likelihood of a return on their investment. As a consequence, when having a relatively small production potential, they intentionally decided not to invest.

Land resources owned by a farm are largely decisive for its competitive position and capacity to generate incomes. Both absolute and relative values are important, especially including how they compare to other farms in the country or region concerned. For reasons referred to above (i.e. a nationalized economy in the socialist era and the dominant privatization path), the majority of farms covered by the FADN (i.e. farms which jointly contribute 90% to standard output) are large farms with an average area above 100 ha (Table 2) in most countries surveyed, the largest being found in Bulgaria and Hungary. The average farm area is definitely smaller in Poland and Slovenia, i.e. countries whose economic history followed a slightly different path when it comes to these aspects. In each case, underinvested farms are the ones with the smallest area; this is true for both periods (2007–2009 and 2013–2015). Also, all countries in this group, except for Slovenia, saw a decline in the average farm area (the sharpest being recorded in Estonia). This suggests that managers of relatively small farms failed to examine their competitive advantages (as described above); they gradually withdraw from farming by reducing investments and selling out their land. Note that what matters in this context is the relative size compared to other farms based in the country concerned. Underinvested farms in Bulgaria, Estonia, Hungary and Latvia are much larger than Polish and Slovenian farms at optimum investment levels. In turn, the largest holdings are relatively overinvested farms (Bulgaria, Latvia, Poland) or absolutely overinvested farms (Hungary, Slovenia). Only in Estonia, the largest farms were found in the group at optimum investment levels. Most farms (except for underinvested ones) saw an increase in their average area, with the highest growth rates being usually recorded by relatively overinvested holdings. Interestingly, farms at optimum investment levels witnessed a small increase in acreage, which could mean they mostly embarked on a capital-intensive development path.

**Table 2:** Land resources (ha per farm) in farms grouped by scale of investment in 2007–2009 and 2013–2015 (*I*_*8*_).

Farms	Bulgaria			Estonia			Hungary			Latvia			Poland			Slovenia		
	${\bar{I}}_{1_{2007-2009}}$	${\bar{I}}_{1_{2013-2015}}$	Δ*I*_1_	${\bar{I}}_{1_{2004-2006}}$	${\bar{I}}_{1_{2013-2015}}$	Δ*I*_1_	${\bar{I}}_{1_{2007-2009}}$	${\bar{I}}_{1_{2013-2015}}$	Δ*I*_1_	${\bar{I}}_{1_{2007-2009}}$	${\bar{I}}_{1_{2013-2015}}$	Δ*I*_1_	${\bar{I}}_{1_{2007-2009}}$	${\bar{I}}_{1_{2013-2015}}$	Δ*I*_1_	${\bar{I}}_{1_{2007-2009}}$	${\bar{I}}_{1_{2013-2015}}$	Δ*I*_1_
Absolute overinvestment	333.5	347.0	13.5	168.7	190.4	21.7	336.6	299.9	-36.7	183.5	226.9	43.4	36.5	43.9	7.4	22.5	23.2	0.7
Relative overinvestment	583.5	545.0	-38.5	219.1	280.6	61.5	258.6	280.3	21.7	430.8	459.5	28.7	35.9	49.9	14.0	14.1	16.4	2.3
Underinvestment	209.4	185.3	-24.1	137.6	89.0	-48.6	149.8	121.0	-28.8	64.0	58.3	-5.7	20.4	19.9	-0.5	12.0	12.2	0.2
Optimum	350.1	326.3	-23.8	429.8	473.4	43.6	192.0	194.9	2.9	339.8	408.0	68.2	32.5	37.5	5.0	15.8	17.0	1.2

Source: own calculations based on unpublished EU-FADN–DG AGRI data.

In each country, farm labor resources (Table 3) depend both on the average farm area and on the type of activity. In both periods covered by this study, the amounts of labor were by far the largest in Bulgaria which can be explained by the dominance of a labor-intensive horticulture. A similar situation was found in Hungary (especially in the ‘absolute overinvestment’ group) and in Latvia (in the ‘relative overinvestment’ group). In other cases, labor resources are at a level typical of individual farms where hired labor is only used to supplement the farming family’s own labor resources. Just like in the case of land, the smallest labor resources are found in underinvested farms. Furthermore, underinvested farms are the ones that experienced the greatest decline in labor resources which, once again, can suggest they gradually discontinue their farming activities (the sharpest reduction was recorded in Estonia and Hungary). Nevertheless, a reduction in labor resources was observed in each country and in nearly every group. Bulgarian farms from the ‘absolute overinvestment’ group were the only ones to report an increase (by 1.1 AWU). In most countries, the sharpest decline was experienced in the ‘relative overinvestment’ group. The trend towards reducing labor resources is generally typical of today’s modern agriculture characterized by the substitution of labor for capital. However, the change in relationships between labor and capital differs on a case-by-case basis and depends on the particularities of each farm. Indeed, the purposes of investing include an improvement in labor productivity which can be achieved without changes (or even with an increase) in employment [41].

**Table 3:** Labor resources (AWU per farm) in farms grouped by scale of investment in 2007–2009 and 2013–2015 (*I*_*2*_).

Farms	Bulgaria			Estonia			Hungary			Latvia			Poland			Slovenia		
	${\bar{I}}_{2_{2007-2009}}$	${\bar{I}}_{2_{2013-2015}}$	Δ*I*_2_	${\bar{I}}_{2_{2004-2006}}$	${\bar{I}}_{2_{2013-2015}}$	Δ*I*_2_	${\bar{I}}_{2_{2004-2006}}$	${\bar{I}}_{2_{2013-2015}}$	Δ*I*_2_	${\bar{I}}_{2_{2004-2006}}$	${\bar{I}}_{2_{2013-2015}}$	Δ*I*_2_	${\bar{I}}_{2_{2004-2006}}$	${\bar{I}}_{2_{2013-2015}}$	Δ*I*_2_	${\bar{I}}_{2_{2004-2006}}$	${\bar{I}}_{2_{2013-2015}}$	Δ*I*_2_
Absolute overinvestment	10.8	11.9	1.1	2.9	2.3	-0.6	10.6	9.1	-1.5	4.7	4.9	0.2	2.1	2.2	0.1	2.4	1.9	-0.5
Relative overinvestment	17.8	16.1	-1.7	5.4	5.3	-0.1	6.5	6.4	-0.1	15.8	14.2	-1.6	2.2	2.5	0.3	9.3	8.4	-0.9
Underinvestment	14.9	10.3	-4.6	2.4	1.1	-1.3	6.0	4.5	-1.5	2.2	1.5	-0.7	1.8	1.7	-0.1	1.6	1.4	-0.2
Optimum	17.1	13.2	-3.9	9.9	7.3	-2.6	4.5	4.3	-0.2	9.9	8.6	-1.3	2.2	2.1	-0.1	1.8	1.7	-0.1

Source: own calculations based on unpublished EU-FADN–DG AGRI data.

The differences in capital resources between countries are similar to what was found with respect to land. In this context, note that Polish and Slovenian farms generally have a lower value of fixed assets. The attribution of farms to corresponding groups largely depended on changes in capital resources, and therefore it is obvious that the greatest increments in that productive input are recorded either in the ‘relative overinvestment’ or in the ‘absolute overinvestment’ group (Table 4). In these groups, the growth rate for capital is (often much) greater than the growth rate for land and labor. Capital resources more than doubled in each farm of the ‘relative overinvestment’ group. Obviously, in each country, underinvested farms have the smallest capital resources which always continue to decline. Once again, this suggests they gradually discontinue their agricultural activity. All other groups experienced an increase, with the smallest growth being recorded by farms at optimum investment levels (which is largely consistent with the metrological assumptions made in this study). The different investment strategies can only be evaluated in a context of economic effects they attain (as discussed in more detail later in this paper). Nevertheless, if account is taken only of the results shown in Table 3 and of the methodological assumptions of this paper, it can be noticed that as regards the ‘absolute overinvestment’ group, the increase in capital resources (which was usually considerable) was unreasonable as it failed to translate into improvements in labor productivity. Things look slightly different in the ‘relative overinvestment’ group: the increase in capital resources was so sharp that the increase in labor productivity could not keep pace with it. This means that either some of the investments were useless or such a considerable increase in capital was necessary to attain a specific improvement in productivity. In the latter case, the availability of EU funds would rather provide an opportunity for improvements in economic performance which otherwise would be impossible or difficult to reach. Note that the changes experienced in that group of farms largely reflect the general characteristics of agriculture, including a great demand for, and underutilization of, fixed assets in the form of machinery and buildings (as field machinery is only used in the growing season), and a relatively low production profitability. Farms at optimum investment levels need to be assessed in that very context. The fact alone that labor productivity grew faster than capital is certainly a significant advantage which means they followed a development strategy that makes efficient use of inputs other than only capital. This could include improvements in farming efficiency based on the adoption of state-the-art techniques and technologies; improvements in production organization; integration; adequate marketing strategies; or, last but not least, improvements in human capital which accompany the investment process. This is all the more possible since, in most countries covered by this study, these are not the largest farms and therefore it would be reasonable for them to rely on more intensive production methods. When it comes to underinvested farms, all of their characteristics (and, most notably, the trends they follow) suggest they gradually discontinue their agricultural operations. The absence of a competitive edge substantiates their decision. However, irrespective of what drives this process, the performance figures for their productive inputs suggest that refraining from investing inevitably leads to a decline in production potential, manifested by a reducing value of capital and land (and labor, too). Therefore, the situation of underinvested farms should rather be evaluated in a context of multipurpose rural development which, however, goes beyond the scope of this paper. However, it is worth discussing the problem of land resources owned by these farms. Professional mobility and the ability to find a non-agricultural job would result in releasing land to the market. As a consequence, land would be acquired by farms which invest and develop, and could improve their economic performance. This primarily means farms at optimum investment levels and members of the ‘relative overinvestment’ group. In this situation, current economic decisions on the scale of investment become a determinant of structural changes in agricultural inputs (which are anyway necessary in most CEE countries).

**Table 4:** Resources of fixed assets other than land (EUR thousand per farm) in farms grouped by scale of investment in 2007–2009 and 2013–2015 (*I*_*3*_).

Farms	Bulgaria			Estonia			Hungary			Latvia			Poland			Slovenia		
	${\bar{I}}_{3_{2007-2009}}$	${\bar{I}}_{3_{2013-2015}}$	Δ*I*_3_	${\bar{I}}_{3_{2004-2006}}$	${\bar{I}}_{3_{2013-2015}}$	Δ*I*_3_	${\bar{I}}_{3_{2004-2006}}$	${\bar{I}}_{3_{2013-2015}}$	Δ*I*_3_	${\bar{I}}_{3_{2004-2006}}$	${\bar{I}}_{3_{2013-2015}}$	Δ*I*_3_	${\bar{I}}_{3_{2004-2006}}$	${\bar{I}}_{3_{2013-2015}}$	Δ*I*_3_	${\bar{I}}_{3_{2004-2006}}$	${\bar{I}}_{3_{2013-2015}}$	Δ*I*_3_
Absolute overinvestment	305.3	580.1	274.8	130.6	299.2	168.6	638.5	1055.4	416.9	169.3	471.0	301.7	118.8	200.9	82.1	116.3	188.9	72.6
Relative overinvestment	458.5	1015.3	556.8	259.3	681.1	421.8	346.3	813.7	467.4	492.2	1758.8	1266.6	120.6	255.6	135.0	137.1	377.9	240.8
Underinvestment	405.1	256.4	-148.7	120.4	82.7	-37.7	362.3	295.9	-66.4	54.8	40.7	-14.1	79.4	61.2	-18.2	71.5	54.5	-17
Optimum	507.2	749.3	242.1	620.5	904.7	284.2	314.3	420.5	106.2	361.0	753.0	392.0	100.0	124.7	24.7	119.1	184.7	65.6

Source: own calculations based on unpublished EU-FADN–DG AGRI data.

Production efficiency is conditioned by factors which include appropriate relationships between productive inputs, such as the assets-to-land and assets-to-labor ratios. In this context, note that both the excessively low and excessively high levels reduce the farm’s ability to maximize economic performance. In the former case, this is because capital deficiencies reduce the use intensity of other productive inputs and restrict the production capacity defined as both the quantity and quality of final output. In the latter case, an excessive amount of capital in relation to other productive inputs (in agriculture, this mainly means land) adds to costs while not increasing the production scale.

In most countries covered by this study, the assets-to-land ratio (Table 5) reached the highest levels in the ‘absolute overinvestment’ group or the ‘relative overinvestment’ group (more frequently). Also, these are the groups which experienced the greatest extent of changes (the ratio more than doubled in many cases). It is difficult to clearly assess whether such an in-depth transformation is justified. In the first case, this could mean that in the base period, the assets-to-land ratio was excessively low in relation to the production potential, and therefore the changes would be justified. However, the second case could mean overinvestment, i.e. a process whereby capital resources are expanded beyond the productive capacity of land. In accordance with the methodological assumptions adopted in this paper, the second scenario is true. However, as already mentioned, it should be assessed differently depending on whether it took place in the ‘absolute overinvestment’ or the ‘relative overinvestment’ group. Indeed, the particularities of natural and technical conditions of agricultural production could result in the capital growth rate (and the growth rate of the assets-to-land ratio analyzed in this paper) being much higher than growth in production volumes or improvements in economic performance. Obviously, positive growth of capital accompanied by a decline in labor productivity (as it is the case in the ‘absolute overinvestment’ group) is a symptom of manifest errors in both investments and operations. An abnormal situation took place in Bulgaria, Hungary and Poland: in the initial period of this analysis, the highest value of the assets-to-land ratio was recorded in underinvested farms. In these (and other) countries, the assets-to-land ratio declined in that group, and so did other parameters. The largest drop was witnessed in Slovenia and Poland. Estonia was the only country where that ratio remained virtually unchanged. However, it is difficult to view that process as a rationalization of relationships between land and capital resources, for instance because the corresponding ratios in that group (except for Bulgaria, Hungary and Poland, as mentioned earlier) were usually at the lowest levels already in the first years of this analysis. Hence, the decline in that ratio rather seems to corroborate the supposed gradual discontinuation of farming activities.

**Table 5:** Value of fixed assets other than land, calculated per hectare of agricultural land (EUR/ha), in farms grouped by scale of investment in 2007–2009 and 2013–2015 (*I*_*4*_).

Farms	Bulgaria			Estonia			Hungary			Latvia			Poland			Slovenia		
	${\bar{I}}_{4_{2007-2009}}$	${\bar{I}}_{4_{2013-2015}}$	Δ*I*_4_	${\bar{I}}_{4_{2004-2006}}$	${\bar{I}}_{4_{2013-2015}}$	Δ*I*_4_	${\bar{I}}_{4_{2004-2006}}$	${\bar{I}}_{4_{2013-2015}}$	Δ*I*_4_	${\bar{I}}_{4_{2004-2006}}$	${\bar{I}}_{4_{2013-2015}}$	Δ*I*_4_	${\bar{I}}_{4_{2004-2006}}$	${\bar{I}}_{4_{2013-2015}}$	Δ*I*_4_	${\bar{I}}_{4_{2004-2006}}$	${\bar{I}}_{4_{2013-2015}}$	Δ*I*_4_
Absolute overinvestment	484.6	927.1	442.5	551.0	1118.3	567.0	977.4	2037.8	1060.4	483.1	1418.7	935.6	2539.7	3558.3	1018.6	4707.5	6681.7	1974.2
Relative overinvestment	366.5	831.7	465.2	832.4	1720.7	888.3	622.3	1319.2	696.9	567.4	2396.3	1828.9	2621.0	3933.2	1312.2	8817.7	19505.4	10687.7
Underinvestment	935.1	798.4	-136.7	673.2	667.9	-5.3	1370.8	1320.4	-50.4	416.4	358.9	-57.5	3169.1	2274.0	-895.1	5257.8	3436.6	-1821.2
Optimum	825.6	1271.7	446.1	1134.8	1253.8	119.0	920.9	1115.1	194.2	533.4	1158.2	624.8	2402.7	2351.6	-51.1	7074.8	9150.6	2075.8

Source: own calculations based on unpublished EU-FADN–DG AGRI data.

The potential of today’s agriculture largely depends on the use of capital and of state-of-the-art technologies. Therefore, the higher the assets-to-labor ratio, the greater the competitiveness of a farm. In reality, the inhibiting factor are land resources which either do or do not enable an efficient use of capital owned. This is why in most countries surveyed (Table 6), the highest levels of and the greatest increments in the assets-to-land ratio are found in the ‘relative overinvestment’ or the ‘absolute overinvestment’ group. In Estonia and Hungary, these ratios were at the lowest level in both groups in the base period. However, they reached the highest levels in 2013–15 as a consequence of investments. The situation is different in Bulgaria and Slovenia: in both periods, the highest values of the assets-to-labor ratio were recorded in farms at optimum investment levels. However, the changes were relatively minor which could suggest these farms were highly reasonable in their investment activities. Note also that in most countries, underinvested farms reported the lowest values of that ratio; however, it followed a slight upward trend (except for Poland and Slovenia) driven by the reduction in labor force being faster than the reduction in capital rather than by the implementation of investments (Tables 3 and 4).

**Table 6:** Value of fixed assets other than land, calculated per employee (EUR/AWU), in farms grouped by scale of investment in 2007–2009 and 2013–2015 (*I*_*5*_).

Farms	Bulgaria			Estonia			Hungary			Latvia			Poland			Slovenia		
	${\bar{I}}_{5_{2007-2009}}$	${\bar{I}}_{5_{2013-2015}}$	Δ*I*_5_	${\bar{I}}_{5_{2004-2006}}$	${\bar{I}}_{5_{2013-2015}}$	Δ*I*_5_	${\bar{I}}_{5_{2004-2006}}$	${\bar{I}}_{5_{2013-2015}}$	Δ*I*_5_	${\bar{I}}_{5_{2004-2006}}$	${\bar{I}}_{5_{2013-2015}}$	Δ*I*_5_	${\bar{I}}_{5_{2004-2006}}$	${\bar{I}}_{5_{2013-2015}}$	Δ*I*_5_	${\bar{I}}_{5_{2004-2006}}$	${\bar{I}}_{5_{2013-2015}}$	Δ*I*_5_
Absolute overinvestment	15.0	27.2	12.2	32.1	93.9	61.8	31.2	67.0	35.8	18.8	66.2	47.4	44.5	70.6	26.1	44.3	83.1	38.8
Relative overinvestment	12.0	28.2	16.2	34.1	90.8	56.7	24.7	57.4	32.7	15.5	77.4	61.9	42.2	78.6	36.4	13.3	38.2	24.9
Underinvestment	13.3	14.4	1.1	38.7	54.0	15.3	34.3	35.3	1.0	12.0	14.2	2.2	35.7	26.6	-9.1	38.4	30.9	-7.5
Optimum	16.9	31.5	14.0	49.4	81.5	32.1	39.0	51.1	12.1	18.3	55.1	36.8	35.9	42.5	6.6	61.2	93.1	31.9

Source: own calculations based on unpublished EU-FADN–DG AGRI data.

It is difficult to estimate the optimum relationships between productive inputs as they depend on a number of aspects, e.g. activity type (a more intensive activity intrinsically requires greater amounts of capital), cyclical conditions (price relationships) and natural factors (climate and soil conditions). Furthermore, a major role is played by non-measurable factors specific to each farm, such as skills, knowledge and experience of the manager; risk management; production integration, diversification or specialization; particularities of the local market; and the social and economic environment. Nevertheless, the status of and changes in the relationships between productive inputs can be primarily assessed in the context of production and economic performance of a farm or a group of farms.

Production and economic performance figures are the ultimate validation of investment decisions. Indeed, it has to be assumed that investment activities are an auxiliary function to operations because the objective of each enterprise (including farms) is to maximize economic value. In turn, from the social point of view of today’s expectations, agriculture requires more and more capital resources and innovative solutions in order to meet the challenge of feeding a growing population, especially as only a small proportion of workforce are employed in agriculture in highly developed countries. However, this general conclusion is not always reflected in farm-level realities. As mentioned earlier, at a micro level, the basic barrier to a reasonable use of capital is the area of utilized land. This is why excessive (unfounded) investments can often lead to stagnation in production and to a deterioration in economic performance. Obviously, the consequences of a lack of investments can be similar or even worse. In the countries covered by this study (Table 7), production grows in all groups except for underinvested farms (only Slovenia recorded a growth of EUR 2,700). This is the consequence of both investment activities and improvements in business conditions after the accession to the EU. In most countries, just like in the case of other ratios, the highest values and the greatest changes were recorded in the ‘relative overinvestment’ group (which is all the more understandable since these are usually the largest farms). However, the characteristic feature of this group is that capital grows faster than production. A similar situation occurs in the ‘absolute overinvestment’ group. Abnormal patterns can be found in Hungary where the greatest production volumes are recorded in the ‘absolute overinvestment’ group, and in Estonia where farms at optimum investment levels report the greatest production volumes.

**Table 7:** Production value (EUR thousand per farm) in farms grouped by scale of investment in 2007–2009 and 2013–2015 (*I*_*6*_).

Farms	Bulgaria			Estonia			Hungary			Latvia			Poland			Slovenia		
	${\bar{I}}_{6_{2007-2009}}$	${\bar{I}}_{6_{2013-2015}}$	Δ*I*_6_	${\bar{I}}_{6_{2004-2006}}$	${\bar{I}}_{6_{2013-2015}}$	Δ*I*_6_	${\bar{I}}_{6_{2004-2006}}$	${\bar{I}}_{6_{2013-2015}}$	Δ*I*_6_	${\bar{I}}_{6_{2004-2006}}$	${\bar{I}}_{6_{2013-2015}}$	Δ*I*_6_	${\bar{I}}_{6_{2004-2006}}$	${\bar{I}}_{6_{2013-2015}}$	Δ*I*_6_	${\bar{I}}_{6_{2004-2006}}$	${\bar{I}}_{6_{2013-2015}}$	Δ*I*_6_
Absolute overinvestment	237.7	350.6	112.9	70.3	130.9	60.6	433.8	583.8	150.0	95.8	203.2	107.4	47.2	75.0	27.8	23.3	43.3	20.0
Relative overinvestment	477.6	745.2	267.6	151.1	383.7	232.6	251.0	433.3	182.3	315.4	818.9	503.5	52.4	114.9	62.5	68.3	172.3	104.0
Underinvestment	190.8	175.3	-15.5	48.7	27.6	-21.1	207.3	171.5	-35.8	30.9	25.0	-5.9	26.9	26.4	-0.5	12.9	15.6	2.7
Optimum	325.6	645.9	320.3	274.4	554.5	280.1	159.8	249.7	89.9	191.3	407.0	215.7	37.9	69.2	31.3	25.0	59.1	34.1

Source: own calculations based on unpublished EU-FADN–DG AGRI data.

Once completed, investments can contribute to either a reduction or an increase in costs. The use of more advanced and more efficient machinery and equipment can help making production less energy-intensive, e.g. by reducing the number of runs or by using more fuel-efficient engines. However, the fact alone of having additional fixed assets generates costs such as depreciation, insurance or repair. Moreover, the changes in cost levels should go hand in hand with changes in production levels, ultimately leading to improvements in economic performance. In most cases covered by this study, the ‘relative overinvestment’ and ‘absolute overinvestment’ groups (Table 8) reported the largest amounts of and the highest growth rates for total costs, except for Estonia where the highest costs were incurred by farms at optimum investment levels. In all countries, underinvested farms had the smallest costs and the lowest growth rates. Generally, the amount of total costs was on an upward trend. This could result both from the increase in prices of basic productive inputs and from the increased production intensification (leading to a larger scale of production).

**Table 8:** Total production costs (EUR thousand per farm) in farms grouped by scale of investment in 2007–2009 and 2013–2015 (*I*_*7*_).

Farms	Bulgaria			Estonia			Hungary			Latvia			Poland			Slovenia		
	${\bar{I}}_{7_{2007-2009}}$	${\bar{I}}_{7_{2013-2015}}$	Δ*I*_7_	${\bar{I}}_{7_{2004-2006}}$	${\bar{I}}_{7_{2013-2015}}$	Δ*I*_7_	${\bar{I}}_{7_{2004-2006}}$	${\bar{I}}_{7_{2013-2015}}$	Δ*I*_7_	${\bar{I}}_{7_{2004-2006}}$	${\bar{I}}_{7_{2013-2015}}$	Δ*I*_7_	${\bar{I}}_{7_{2004-2006}}$	${\bar{I}}_{7_{2013-2015}}$	Δ*I*_7_	${\bar{I}}_{7_{2004-2006}}$	${\bar{I}}_{7_{2013-2015}}$	Δ*I*_7_
Absolute overinvestment	225.1	417.4	192.3	66.6	158.4	91.9	466.9	665.2	198.3	93.1	231.1	138.0	34.2	69.1	34.8	23.5	53.9	30.4
Relative overinvestment	431.0	728.4	297.4	138.2	389.7	251.5	257.1	411.0	153.8	312.1	826.6	514.5	37.3	86.6	49.3	62.7	143.2	80.5
Underinvestment	187.2	223.3	36.1	46.8	38.9	-7.9	225.1	207.5	-17.6	29.6	31.9	2.3	20.9	24.4	3.5	14.0	22.5	8.5
Optimum	386.7	585.0	198.3	290.5	580.5	289.9	187.2	240.6	53.4	216.6	421.5	204.8	31.1	50.7	19.7	24.3	47.7	23.4

Source: own calculations based on unpublished EU-FADN–DG AGRI data.

They key metrics of viability of investment decisions are the level of and, first of all, the changes in economic performance figures. The performance figures for the base year reflect the resources of productive inputs (primarily land and capital) owned by farms, the skills in managing the production process and finance, and the general condition of the economy. Conversely, the extent of changes indicates the reasonability of the farms’ strategic measures. The differences between farm groups in each country are an aspect of particular importance in this context because they indicate the effect of various (mainly endogenous) factors. Moreover, as the farms covered by this study operate in the EU single market and in the geographic vicinity of Europe, it can also be assumed that—irrespective of their home country—all of them were faced with the same general economic conditions (mainly including price conditions).

Economic performance was measured as Net Value Added (NVA) less operating subsidies (mainly including direct payments; payments under the 2^nd^ pillar of the CAP other than investment support; and national support, if any). NVA itself is the difference between production value and production costs less costs of external inputs (rents, interest on loans, and expenditure on hired labor), adjusted by the balance of taxes and operating payments. Hence, it measures the payment for the use of all productive inputs irrespective of who owns them. As a consequence, it can be used in comparing farms irrespective of whether they operate based on their own or external productive inputs. The amount of subsidies was deducted from NVA because these analyses refer to economic outcomes attained by farms as part of their market activities, and are supposed to indicate the actual efficiency of production processes. The objective of external support is to mitigate the agricultural effects of market failures. However, when taken into account in an analysis, subsidies could distort the true picture of actual outcomes.

In the countries surveyed, the highest NVA levels were recorded in the ‘relative overinvestment’ group. However, the greatest changes were usually witnessed by farms at optimum investment levels (Table 9). In the final period of this analysis (2013–2015), both of these groups had the greatest surplus in each country. Importantly, an increase in NVA was only recorded in that group. In this context, it seems interesting that farms who exhibited a passive attitude towards investments (underinvested farms) as well as those characterized by excessive activity (the ‘absolute overinvestment’ group) ended up with a decline in economic performance. The sole difference between them is that the former have a much greater net value added in the final period. In the case of underinvested farms, this is consistent with the strategy of a gradual discontinuation of farming operations, as described above. However, when it comes to relatively large farms from the ‘absolute overinvestment’ group, this is a symptom of manifest errors in investing which entail certain (often adverse) changes in operations. Another equally interesting and important conclusion from this study is that the relationships discussed above (i.e. an increase in NVA in farms at optimum levels of investment and in the ‘absolute overinvestment’ group) are true for all countries, irrespective of their particularities.

**Table 9:** Net value added (excluding operating subsidies) (EUR thousand per farm) in farms grouped by scale of investment in 2007–2009 and 2013–2015 (*I*_*8*_).

Farms	Bulgaria			Estonia			Hungary			Latvia			Poland			Slovenia		
	${\bar{I}}_{8_{2007-2009}}$	${\bar{I}}_{8_{2013-2015}}$	Δ*I*_8_	${\bar{I}}_{8_{2004-2006}}$	${\bar{I}}_{8_{2013-2015}}$	Δ*I*_8_	${\bar{I}}_{8_{2004-2006}}$	${\bar{I}}_{8_{2013-2015}}$	Δ*I*_8_	${\bar{I}}_{8_{2004-2006}}$	${\bar{I}}_{8_{2013-2015}}$	Δ*I*_8_	${\bar{I}}_{8_{2004-2006}}$	${\bar{I}}_{8_{2013-2015}}$	Δ*I*_8_	${\bar{I}}_{8_{2004-2006}}$	${\bar{I}}_{8_{2013-2015}}$	Δ*I*_8_
Absolute overinvestment	64.9	21.4	-43.41	8.8	-12.0	-20.86	77.3	28.6	-48.71	8.7	-8.7	-17.43	15.7	6.7	-8.97	-1.0	-13.3	-12.23
Relative overinvestment	165.8	231.5	65.72	35.2	63.2	28.04	58.0	103.7	45.67	61.8	135.6	73.77	18.3	32.0	13.69	32.0	75.2	43.21
Underinvestment	58.8	8.1	-50.70	3.1	-9.9	-13.00	33.4	5.7	-27.69	2.5	-5.6	-8.10	6.9	2.4	-4.54	-2.3	-7.3	-5.00
Optimum	18.1	194.5	176.45	29.4	82.6	53.24	12.7	58.9	46.16	-0.5	49.5	50.08	9.2	21.7	12.52	-1.4	11.5	12.96

Source: own calculations based on unpublished EU-FADN–DG AGRI data.

## Discussion

The particularities of each sector have a considerable effect on investment methods. When comparing agriculture to industrial sectors, for instance, it cannot be ignored that industrial investments are both larger and more frequent. This is especially true for the heavy industry where expenses are focused on the industrial infrastructure (e.g. investment in industrial complexes) [42]. The basic barriers to investment include financial restrictions and imperfections of the loan market [43]. Investments are viewed as the driving force for long-term economic growth [44]. The literature relating to investments increasingly emphasizes that, at least in developed countries, agricultural investments are subsidized (which allows to ensure food security), whereas the implementation of state-of-the-art techniques and technologies makes it possible for agricultural activities to have a smaller environmental and climatic impact. Conversely, the lack of investments often leads to decelerated growth of farm productivity [45, 46]; at the same time, obsolete technologies have a considerable adverse impact on the environment.

For these reasons, the need to make agricultural investments in the context of a major systemic shift (including the emergence of a number of technologies designed to improve labor productivity) should not give rise to controversy. Particular focus should be placed on investments in precision farming or, very soon, in the Internet of Things or the blockchain technology which are consistent with Agriculture 4.0, a concept that may affect the way food is produced, processed, traded and, last but not least, consumed [47–49]. Generally, the importance of technological progress in agriculture is measured based on relationships between resources [50]. This means metrics such as the amount of capital per employee, the amount of capital per hectare of agricultural land and the amount of agricultural land per employee.

Overinvestment is a condition where long-term investments are excessively high compared to the production potential (which, in the agriculture, mostly consists of land resources) and ultimately become economically unviable because the costs incurred to buy and maintain assets were higher than production outcomes. The term “overinvestment” itself is more often used in an industrial or corporate context, and the way it affects agriculture is described rarely (despite being a common occurrence). According to some studies, public funds used in addition to conventional inputs also greatly contributed to an increase in agricultural production [51, 52]. This is a finding from macroeconomic research carried out in China. According to the literature, overinvestment (and the resulting excess production) is increasingly often among the reasons behind massive Chinese exports [53–55]. The situation is different at company or farm level: instead of excess production, overinvestment means a decline in economic performance.

Various types of (industrial, agricultural, service) enterprises get over-invested through an irrational use of assets which is intended to increase company value or have a positive effect on financial performance, but is based on an excessively optimistic evaluation of market conditions [56–58]. As a consequence, the expected return on investment projects is below the interest rate offered in capital markets [59, 60]. According to another approach, overinvestment takes place when companies excessively invest in financial or tangible assets [58]. This sometimes happens in the case of external financing (e.g. subsidies) which is intended to be used as specifically indicated by the financing authority. Investment financing decisions should be made by the economic operator concerned, because a relationship exists between external financing and investment decisions [61, 62]. The capital market results in extreme financial restrictions with sub-optimal investment strategies as the ultimate consequence [63]. The financial leverage is another aspect related to corporate overinvestment. If external financing is readily available, as it is the case e.g. in monetary expansion periods, debt is no longer a traditional management control tool whereas excessive systemic liquidity results in the emergence of leverage which encourages investment and, hence, exacerbates the problem of overinvestment [64]. Sustainable investments in both the agricultural and food sector are necessary [65], even though they account for a relatively minor proportion of global capital markets [66].

European agriculture is based on plant production, primarily including the cultivation of cereals and industrial crops [67]. Moreover, countries which are able to produce food for both humans and animals focus their production efforts on meat, meat products and dairy [68]. Before joining the European Union (EU), Central and Eastern European Countries were characterized by a much lower profitability of agriculture than “old” member countries. The accession to the EU was expected to result in an increase in productivity accompanied by a reduction in net agricultural employment [69]. Factors which have an impact on ensuring food security include the Common Agricultural Policy (CAP) implemented in the European Union; one of its initial objectives was to increase agricultural production [70–72]. The CAP was created at a time where food expenses accounted for a large proportion of earnings of the population of member states [73]. Productivity was supposed to improve through measures which include the investment impetus [74]. “Investments in fixed assets,” a measure available under the 2^nd^ pillar of the CAP, is one of the important instruments in this respect. Its objective is to support farm investments and improve the structure of farming land [75]. Major priorities of the CAP after 2020 will include knowledge and technology investments which enable environmental protection and the existence of low-carbon economies [76]. The grounds for agricultural interventionism result from the conditions of the biological nature of agricultural production [77, 78]. Other aspects that substantiate it include informational imperfection, income-related problems of agriculture and the incompleteness and imperfection of markets related to agriculture [79]. Production periodicity and seasonality and economic fluctuations are the reasons why agricultural investment are less effective than in other sectors. Also, the biological growth and development process makes outputs distant in time from inputs while decelerating the movement of capital [21]. Anyway, agricultural subsidies are a guarantee of food security which means that investments should always drive positive production and economic effects. This is particularly true for investment support. In addition to the basic reason (as mentioned above) which is the society’s and government’s concern for an adequate supply of high-quality foods, note also the particular way of using most of agricultural equipment. Unlike industrial machinery or vehicles, agricultural equipment is only used during the growing season which is a few months in most countries around the world. Also, the extent of using it is limited by the size of the farm and, as the case may be, by local demand for agricultural services. As a consequence, field equipment is used for a relatively small part of the year, and therefore the investment and operating expenses per hour of work are much higher than in other sectors. Despite these shortcomings, a farm must own high-performance equipment primarily due to general societal reasons (as it enables efficient production of food by a relative small group of people). However, at a microeconomic level, the farmers lack rationale to purchase it, and thus the need arises for public support.

One of the reason to overinvestment in agriculture is also a lack of trust among farmers. Trust is very important in human relations and cooperation in business relationship, especially in agriculture [80, 81]. The trust in agriculture (often leading to formal integration relationships) has the potential to benefit farmers in gaining access to the innovation and knowledge, increase the productivity, quality and cost reduction. Effective cooperation in agriculture allows, for example, the collective use of machinery, common purchases of production inputs and sales of final products, reduction of transaction costs. Unfortunately, the moral hazard—on the machinery sharing cooperations arrangements as for example—is visible and have negative influence on the cooperation [82]. The moral hazard would be less if all investment subsidies were burdened with individual responsibility [83]. The moral hazard is observed on credit markets [84], including preferential loan in agriculture in CEE. The lack of trust is the most important reason why relationship between farmers do not work well [85]. Poor management of agribusiness establishments can lead to moral hazards that can affect investments in agriculture [86]. The research of Kovacs [87] shows that the performance of Hungarian producer organizations in agriculture is not as developed as in the Western European countries and the moral hazard has an impact on the farmers’ collaboration. The propensity to overinvest is also implied by the propensity to take risks. Overall, low risk inclination among farmers cause under-investment [88]. Every investment comes with a certain amount of risk [89], especially in agriculture. The investment in agriculture is partially financed by public funds often which lowers the risk score of the investment but don’t doesn’t reduce it entirely [90].

## Conclusions

For Central and Eastern European countries, joining the EU was a great leap forward, including in the agricultural sector. Aid funds allocated to farm investments played an important role in that respect, allowing many operators to catch up with progress (some of them dealing with a modernization backlog dating back to the real socialism era). The investments implemented by some farms were excessive in relation to their production potential. Conversely, other ones gradually discontinued their farming operations as they saw no opportunity for gaining a competitive edge.

This study confirmed that investments have a complementary nature vis-à-vis operations. Indeed, production processes require adequate capital resources, whereas economic performance is what ultimately validates investment decisions. This paper used two essential criteria in assessing these aspects: the absolute increase in economic outcomes, and how it relates to the increase in capital resources. This allowed to identify two types of farms, namely the ‘absolute overinvestment’ and ‘relative overinvestment’ groups. The first case is a symptom of manifest errors in investing (and, perhaps, in how operations are conducted), since large amounts of investment expenditure were accompanied by a decline in economic performance. In this context, note that it was not the consequence of unfavorable price relationships because other operators recorded improvements despite dealing with the same market conditions. Things look slightly different in the ‘relative overinvestment’ group. They saw an improvement in economic performance, which is the goal sought by every economic operator. However, the growth was slower than the increase in capital resources. On the one hand, this could suggest excessive investment took place; but on the other hand, these measures could have been required because of the particularities of production. The second interpretation seems more justified as that group exhibited the highest net value added in all countries surveyed (although in practice both scenarios are likely to have taken place).

When assessing the Union program for investment support on an overall basis, it is necessary to exercise extreme caution in the interpretation because some of the relevant decisions were made otherwise than in the context of availability of aid funds. Nevertheless, support from the EU was significant and must be evaluated. Undoubtedly, farms at optimum investment levels should be viewed as being in a positive situation; in their case, economic performance grew faster than capital. In the context of this discussion (although with some reservations presented above), the activity of the ‘relative overinvestment’ group should also be considered a positive development. Despite a decline in most ratios, there was a specific rationale behind the measures taken by small and underinvested farms. Dealing in a specific national context and not having a competitive edge, they gradually discontinued their farming activities. Hence, actual and manifest errors were made in the ‘absolute overinvestment’ group. When assessing the rationality and efficiency of the Union support program, note that the share of this group in the total number of farms ranged from a few to more than ten percent, depending on the country (it went beyond 20% only in Poland). This means that—despite being tempted to abuse the non-repayable aid scheme—most farmers did the right thing and acted responsibly, in accordance with their market position. It could be explained by the fact the farmers contributed their own funds and realized that assets generate costs. The formal barriers to access aid programs and the verification of project plans by the granting institutions could have played a certain role, too.
